# The prognostic value of dynamic changes in SII for the patients with STEMI undergoing PPCI

**DOI:** 10.1186/s12872-023-03679-w

**Published:** 2024-01-23

**Authors:** Ailin Liu, Na Sun, Feiyu Gao, Xiaotong Wang, Hong Zhu, Defeng Pan

**Affiliations:** Department of Cardiology, Affiliated Hospital of Xuzhou Medical University, Xuzhou, China

**Keywords:** Systemic immune-inflammation index, ST-elevation myocardial infarction, Primary percutaneous coronary intervention, Dynamic changes, Prognosis

## Abstract

**Background:**

Predicting the prognosis of primary percutaneous coronary intervention(PPCI) in ST-segment elevation myocardial infarction (STEMI) patients in the perioperative period is of great clinical significance. The inflammatory response during the perioperative period is also an important factor. This study aimed to investigate the dynamic changes in the systemic immune inflammatory index (SII) during the perioperative period of PPCI and evaluate its predictive value for in-hospital and out-of-hospital outcomes in patients with STEMI.

**Methods:**

This retrospective study included 324 consecutive patients with STEMI who were admitted to the cardiac care unit. Blood samples were collected before PPCI, 12 h (T1), 24 h, 48 h after PPCI, the last time before hospital discharge (T2), and 1 month after hospital discharge. The SII was calculated as (neutrophils×platelets)/lymphocytes. Based on whether the primary endpoint occurred, we divided the patients into event and non-event groups. Univariate and multivariate logistic regression analyses were performed to identify independent risk factors that might influence the occurrence of the primary endpoint. Dynamic curves of SII were plotted, and receiver operating characteristic (ROC) curves were drawn for each node to calculate the optimal critical value, sensitivity, and specificity to assess their predictive ability for in-hospital and out-of-hospital courses. Kaplan-Meier curves were used to analyze the differences in survival rates at different SII inflammation levels.

**Results:**

High levels of SII were individually related to the occurrence of the in-hospital period and long-term outcomes during the post-operative follow-up of STEMI patients (in-hospital SII: T1:*OR 1.001,95%CI 1.001–1.001, P < 0.001*; SII following hospital discharge: T1M: *OR 1.008,95%CI 1.006–1.010, P < 0.001*). Patients with high SII levels had lower survival rates than those with low SII levels. The analysis showed that the SII 12 h after (T1) and SII 1 month (T1M) had excellent predictive values for the occurrence of in-hospital and out-of-hospital outcomes, respectively (*AUC:0.896, P < 0.001; AUC:0.892, P < 0.001*).

**Conclusion:**

There is a significant relationship between the dynamic status of SII and prognosis in patients with STEMI. This study found that the 12 h and SII 1 month affected in-hospital and out-of-hospital outcomes, respectively. Consequently, we focused on the dynamic changes in the SII.

**Supplementary Information:**

The online version contains supplementary material available at 10.1186/s12872-023-03679-w.

## Background

Acute ST-segment elevation myocardial infarction (STEMI) is a severe form of coronary atherosclerosis. It is associated with several serious complications, poor prognosis, and high mortality. Primary percutaneous coronary intervention (PPCI) is the most effective reperfusion strategy for the treatment of acute STEMI and can significantly improve prognosis in patients with acute myocardial infarction. However, despite significant progress in revascularization, adverse cardiovascular events still occur during the perioperative period. The in-hospital mortality rate of patients with STEMI remains at 4–12% and the mortality in the first year is still 10% [1]. Therefore, predicting the prognosis of PPCI in patients with STEMI during the perioperative period is clinically significant.

As we all know, several factors influence the prognosis of PPCI in STEMI patients, such as the elderly, the history of hypertension and myocardial infarction, Killip classification, degree of coronary artery disease, etc. [2, 3]. The inflammatory reaction is involved in the pathological process of atherosclerosis and plays an important role in the onset, development, and progression of acute myocardial infarction and the emergence of complications. Inflammation and oxidative stress lead to plaque rupture and consequent atherosclerotic thrombosis, inducing the appearance of acute myocardial infarction symptoms and adverse cardiovascular events [4, 5]. The inflammatory response during the perioperative period is an important factor that has attracted increasing attention. Therefore, the inflammatory response status during the perioperative period in patients with STEMI warrants attention.

Inflammatory biomarkers such as leukocytes, adhesion molecules, and cytokines have been used to study the status of the inflammatory response. Several inflammatory marker ratios, such as the neutrophil/lymphocyte ratio (NLR), lymphocyte/monocyte ratio (LMR), and platelet/lymphocyte ratio (PLR), are associated with the presence and severity of coronary artery disease (CAD) and may predict future coronary events and mortality [6]. NLR is associated with the degree of patency of infarct-related arteries (IRA) before PCI, no reflow after PCI, and cardiac mortality in patients with acute STEMI [7]. PLR is an independent predictor of cardiovascular events and mortality in patients with STEMI [8].

Recently, a new marker of the inflammatory response, the Systemic Immune Inflammation Index (SII), derived from a combination of circulating neutrophils, platelets, and lymphocytes, which was initially useful as a predictor of clinical outcomes in oncology and other inflammatory diseases [9, 10], has now been investigated in cardiovascular diseases. The SII has been shown to be a potential predictor of major cardiovascular and cerebrovascular events and all-cause mortality after PCI in elderly patients of acute myocardial infarction (AMI) [11] and has been associated with the development of contrast nephropathy [12] and the emergence of the no-reflow phenomenon in patients with AMI [13]. Compared to NLR and PLR, SII represents three important immune response pathways, namely the inflammatory response, thrombosis, and stress response, which can reflect the state of the patient’s condition more comprehensively and therefore can be considered a more sensitive indicator of the inflammatory response in the body.

However, the prognostic value in previous studies is limited by the fact that they collected SII levels at baseline as a single item, reflecting only the immediate inflammatory status of patients on admission. The inflammatory response is a dynamic process during the perioperative period of PPCI in patients with STEMI, and a single item alone does not truly reflect the inflammatory process in patients. Serial changes in the SII must be observed. Therefore, we sought to determine whether dynamic changes in the SII during the perioperative period of PPCI in patients with STEMI were related to the development of intra- and extra-hospital cardiovascular outcomes.

## Methods

### Study population

**Fig. 1:**
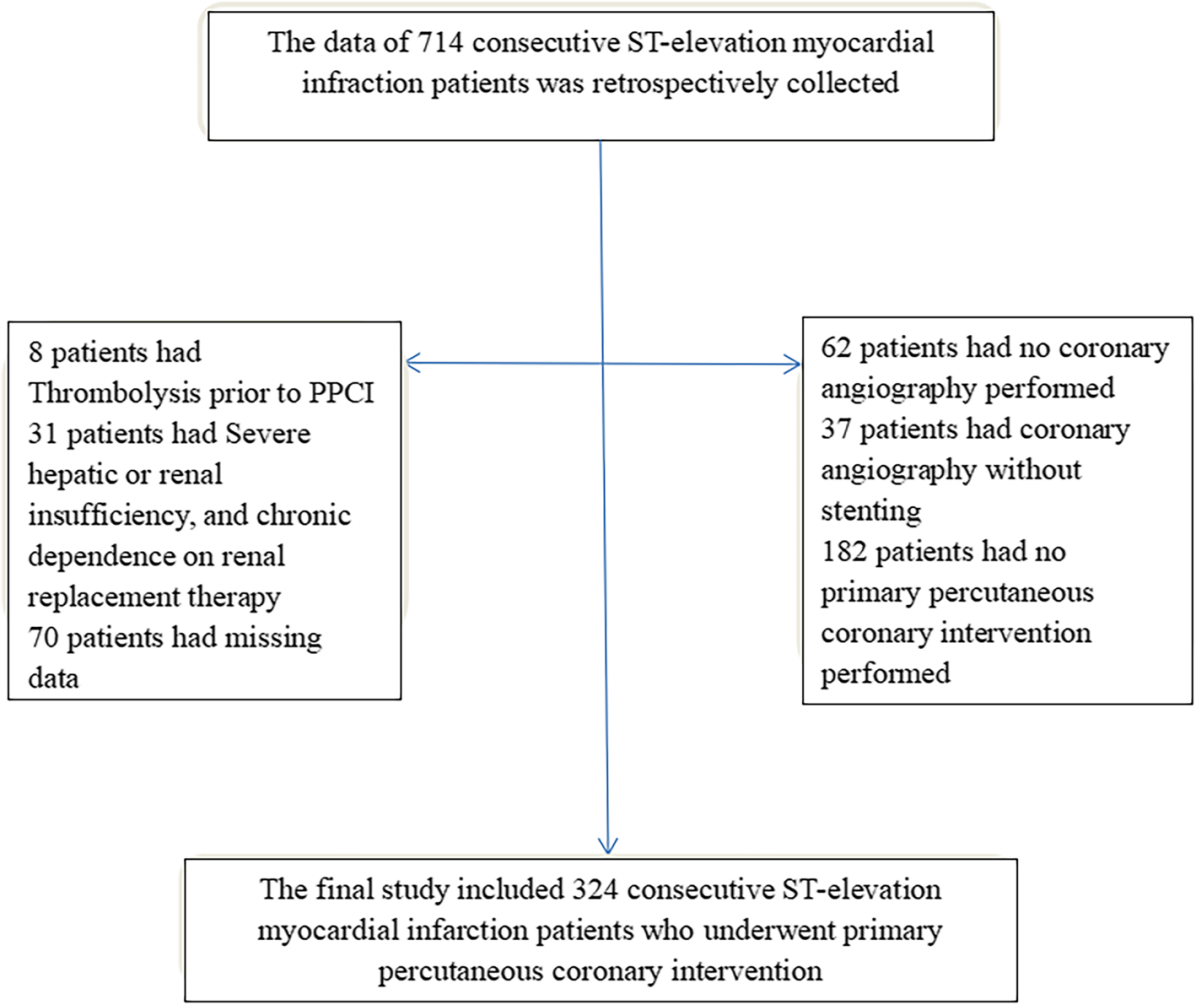
Flow chart of the study participants. Abbreviations: PPCI, primary percutaneous coronary intervention

In the Affiliated Hospital of Xuzhou Medical University between 2019.01.01 and 2021.12.31, 713 patients with acute STEMI in the cardiac care unit were evaluated. Finally, 324 patients met the inclusion criteria (Fig. 1). The study was approved by the hospital ethics committee (XYFY2022-KL420–01).

The collection of clinical data included general and laboratory data.

General data included gender, age, previous history of hypertension, diabetes, stroke/TIA, myocardial infarction /PCI/CABG, coronary artery disease, heart failure, smoking, and alcohol consumption; medications during hospitalization (aspirin, clopidogrel/Ticagrelor, CCB, statins, β-blockers, ACEI/ARB, diuretics), systolic and diastolic blood pressure on admission, heart rate on admission, and Killip classification. The data collected during PPCI included main diseased vessels (left coronary artery, left anterior descending coronary artery, left circumflex coronary artery, and right coronary artery), the total length of the implanted stent, the average diameter of the implanted stent, the number of implanted stents, type of PCI, IVUS assist, TIMI after PPCI, site of AMI, medications discharge (aspirin, P2Y12 receptor inhibitors, diuretics, statins, beta-blockers, ACEI/ARB, insulin), and Chest pain period (h), Pain-to-balloon time (h), Door-to-balloon time (h), hospitalization day, etc.

Laboratory data included hemoglobin, glycated hemoglobin, fasting glucose, glomerular filtration rate, serum creatinine, serum uric acid, AST, ALT, albumin, Troponin T, N-terminal pro-brain natriuretic peptide (NT-proBNP) on admission, monitor blood lipids, including low-density lipoprotein cholesterol (LDL-C), high-density lipoprotein cholesterol (HDL-C), triglycerides, total cholesterol, lipoprotein-a (Lp(a)) on admission, white blood cell count, neutrophil count, monocyte count, lymphocyte count, platelet count, high-sensitivity C-reactive protein level (hs-CRP), cTnI_max_ and CK-MB_max_, etc.

Inclusion criteria: Primary percutaneous coronary intervention performed within 12 hours of symptom onset according to the 2017 STEMI treatment guidelines [14].

Exclusion criteria: (1) Thrombolysis before PPCI, (2) Chronic dependent renal replacement therapy, (3) Glomerular filtration rate (eGFR) < 15 ml/(min*1.73 m2) or severe hepatic insufficiency, (4) Primary hematologic disease or active malignancy, (5) Inflammation (febrile disease, autoimmune disease, acute or chronic inflammatory disease, or recent history of infection), (6) Treatment with chronic steroids or NSAIDs, history of organ transplantation, (7) Including a history of allergy to contrast medium, P2Y12 receptor inhibitors, angiotensin-converting enzyme inhibitors (ACEI), angiotensin II receptor blockers (ARB), aspirin, statins, beta-blockers, etc., (8) Thyroid function disease, heart valve disease, etc. (9) Pregnant or lactating patients.

### Definitions and endpoints

Definition of STEMI: (1) when typical chest pain lasts more than 30 minutes, (2) ST-segment elevations at least at two consecutive leads (V2-V3 at least 0.2 mV for men; 0.15 mV for women and/or 0.1 mV for other leads). (3) Apparent or probable new-onset left bundle branch block; (4) V3R - V4R and V7–V9 leads with an elevation in the ST-segment obtained [1].

Hypertension: Systolic blood pressure > 140 mmHg and/or diastolic blood pressure > 90 mmHg on at least two measurements, or the use of any anti-hypertensive medication [15].

Diabetes mellitus: Fasting plasma glucose level > 7 mmol/L or > 11.1 mmol/L on any measurement, any anti-diabetic drug use, or HbA1c ≥6.5% [15].

Hyperlipidemia: Total cholesterol > 5.2 mmol/L, low-density lipoprotein cholesterol (LDL-C) > 3.4 mmol/L, triglyceride > 1.7 mmol/L, or anti-lipid treatment [15].

Smokers: Currently smoked or quit smoking within the last year [15].

Drinkers: Currently drink or quit drinking within the last year [15].

Follow-up Period: Clinical data of the patients during hospitalization and by review at the clinic or telephone follow-up discharge. The follow-up period at discharge was 12 months.

The main endpoints were as follows.

The hospitalization outcomes included cardiogenic shock, acute respiratory failure, acute renal injury, ventricular arrhythmia, recurrent infarction, vascular reconstruction (PCI/CABG), and all-cause death.

The cardiovascular outcomes (12 months follow-up) included all-cause death, recurrent infarction, revascularization (PCI/CABG), and ventricular arrhythmia.

Before administering any medication, an anterior venous blood sample was drawn from the elbow of each patient in the emergency department on admission. Complete blood counts, including platelet, total leukocyte, neutrophil, and lymphocyte counts, were collected using a full blood count analyzer (Sysmex XT-1800, Sysmex Corporation). Biochemical parameters, such as blood urea nitrogen (BUN), serum creatinine, uric acid, lipid profile, and cardiac enzyme levels, were obtained in our central laboratory using an auto-analyzer (Hitachi 747, Tokyo, Japan).

Peripheral blood was collected from STEMI patients who met the inclusion criteria before PPCI (T0), within 12 h after (T1), within 24 h after (T24), within 48 h after (T48), within the last time before hospital discharge (T2), and 1 month after hospital discharge (T1M), respectively, and SII levels were calculated.

The SII was calculated as total peripheral platelet count (P) × (neutrophil count/lymphocyte ratio) NLR (N/L) (SII = P × N/L ratio).

All patients included in this study were administered 300 mg chewable preoperative aspirin, ticagrelor 180 mg orally, and unfractionated heparin 70–100 U/kg IV. PCI was performed by experienced physicians qualified for coronary intervention. Primary PCI was performed using a standard radial approach with a 6 or 7 French catheter. The stent type (bare metal or drug-eluting stent) and thrombus aspiration were determined by the operator. Glycoprotein IIb/IIIa receptor inhibitors were selected by the operator and were administered during PCI by intra-coronary push of 10 μg/kg followed by an intravenous infusion of 0.15 μg/kg/min. If necessary, balloon dilatation was performed to ensure stent appositioning. The coronary artery lesions were treated using standard PCI techniques. The standard Judkins technique (Expo; Boston Scientific Corporation, Natick, Massachusetts, USA) and Siemens Axiom Sensis XP device (Munich, Germany) were used for selective coronary angiography. An iopromide contrast agent (Ultravist-370 Schering AG, Berlin, Germany) was used. Medications taken by the patients during hospitalization and after hospital discharge were in accordance with the guidelines of the European Society of Cardiology.

### Statistics and analysis

Baseline characteristics of the patients were categorized according to the occurrence of the primary endpoint. The Kolmogorov-Smirnov test was used to test for normality. The measured normal data were expressed as mean ± standard deviation (x ± s), and the differences between groups were compared with a t-test. The measurement deviation data are expressed as median (interquartile distance), and the M-U test was used to compare the differences between groups. Numerical data are presented as numbers and percentages, and intergroup comparisons were assessed using the chi-square test and Fisher’s exact test. Univariate and multivariate analyses were performed using logistic regression models to analyze risk factors that may influence the occurrence of the primary endpoint. Receiver operating characteristic (ROC) curves were used to assess the ability of the SII to predict in-hospital and out-of-hospital outcomes after PPCI in patients with STEMI. Survival conditions in the two groups were compared using Kaplan-Meier analysis. The overall survival rate from the date of diagnosis to the date of death or last follow-up was calculated to analyze whether high SII levels correlated with the occurrence of the primary endpoints. The log-rank test was used to analyze differences between groups. *P < 0.05* was considered as a statistically significant difference at the *α = 0.05* test level, and the confidence interval (CI) for all hazard ratios was 95%. SPSS 26.0 statistical analysis software, GraphPad Prism (version 7.0) was used for the statistical analysis.

## Results

### General information data

This study included 324 patients with STMEI who underwent PPCI. The patients were divided into event and non-event groups based on the results of follow-up during hospitalization and 12 months after hospital discharge. The clinical and laboratory data were compared between the two groups.

Tables 1 and 2 summarize the demographic and laboratory data and previous treatment information for all patients. Event and non-event groups were divided according to the presence or absence of out-of-hospital outcomes after hospital discharge. The degree of multiple vascular lesions > 50%, number of implanted stents, IVUS-assisted application of P2Y12 receptor inhibitors, LDL-C, NT-proBNP, hs-CRP_max,_ and six groups of SII levels were statistically significant in the event group compared to the non-event group (*P < 0.05*).

**Table 1:** Comparison of characteristacs, medications between after hospital discharge the event and non-the event

	The event(*n = 73*)	non-the event(*n = 251*)	*t/χ2/z*	*P value*
Gender,n(%)				
Male	60(82.19)	191(76.10)	1.204	0.273
Female	13(17.81)	60(23.90)		
Age (years)	64(21)	66(18)	−0.917	0.359
Chest pain period(h)	4(5.00)	5(4.00)	−1.603	0.109
Pain-to-balloon time(h)	5(4.76)	5.5(4.71)	−1.083	0.279
Door-to-balloon time(h)	1(0.41)	1(0.57)	−1.768	0.077
hospitalization day(d)	6(3)	6(2)	−0.355	0.722
History				
Hypertension,n(%)	31(42.47)	104(41.43)	0.025	0.875
Diabetes mellitus,n(%)	11(15.07)	54(21.51)	1.465	0.226
Stroke or TIA,n(%)	7(9.59)	27(10.76)	0.082	0.774
Previous MI/PCI/CABG,n(%)	7(9.59)	12(4.78)	1.577	0.209
Heart Failure,n(%)	4(5.48)	6(2.39)	0.919	0.338
Coronary heart disease,n(%)	11(15.07)	24(9.56)	1.780	0.182
Current smoking status,n(%)	24(32.88)	87(34.66)	0.080	0.777
Current drinking status,n(%)	13(17.81)	47(18.73)	0.032	0.859
Admission medication				
Aspirin,n(%)	73(100.00)	251(100.00)	–	–
Clopidogrel or Tigrigrel,n(%)	73(100.00)	251(100.00)	–	–
ACEIs or ARBs,n(%)	41(56.16)	142(56.57)	0.004	0.950
CCB,n(%)	38(52.05)	113(45.02)	1.125	0.289
Β-blocker,n(%)	69(94.52)	219(87.25)	3.026	0.082
Statin,n(%)	73(100.00)	251(100.00)	–	–
Diuretics,n(%)	31(42.47)	82(32.67)	2.390	0.122
SBP (mmHg)	121(26)	123(29)	−0.385	0.700
DBP (mmHg)	77(15)	77(18)	−0.249	0.804
Heart-rate (beats-per-minute)	82.40 ± 13.34	80.61 ± 14.43	0.003	0.343
Killip class,n(%)				
1–2	65(89.04)	228(90.84)	0.211	0.646
3–4	8(10.96)	23(9.16)		
Target Vessel,n(%)				
LM	4(5.48)	5(1.99)	1.419	0.234
LAD	47(64.38)	144(57.37)	1.149	0.284
LCX	7(9.59)	30(11.95)	0.312	0.576
RCA	18(24.66)	77(30.68)	0.989	0.320
Simultaneous treatment of vascular situations,n(%)				
1	70(95.89)	246(98.01)		
> 1	3(4.11)	5(1.99)	0.357	0.550
Vessel-disease (stenosis > 50%)				
1 vessel,n(%)	21(28.77)	69(27.49)	0.046	0.830
2 vessels,n(%)	25(34.25)	56(22.31)	4.297	0.038^a^
3 vessels,n(%)	27(36.99)	126(50.20)	3.961	0.047^a^
PCI type,n(%)				
Only PTCA	8(10.96)	27(10.76)	0.002	0.961
PTCA and Stent	65(89.04)	224(89.24)		
Stent type,n(%)				
Drug eluting stent	24(32.88)	105(41.83)	1.893	0.169
Bare metal stent	49(67.12)	146(58.17)		
Number of stents inserted,n(%)				
1 piece	61(83.56)	230(91.63)	4.028	0.045^a^
≥2 piece	12(16.44)	21(8.37)		
Stent length (mm)	24(9)	24(10)	−0.209	0.834
Stent Diameter (mm)	3(0.5)	3(0.5)	−0.156	0.876
IVUS Assist,n(%)				
Yes	7(9.59)	6(2.39)	5.855	0.016^a^
No	66(90.41)	245(97.61)		
TIMI after PPCI n(%)				
3	72(98.63)	244(97.21)	0.067	0.795
<3	1(1.37)	7(2.79)		
Site of AMI n(%)				
anterior	42(57.53)	128(51.00)	1.759	0.799
antero-septal	2(2.74)	8(3.19)		
high side wall	1(1.37)	4(1.59)		
Inferior wall	27(36.99)	109(43.43)		
anterolateral	1(1.37)	2(0.80)		
Out-hospital medication,n(%)				
Aspirin	69(94.52)	248(98.80)	3.093	0.079
PY212 receptor inhibitors	68(93.15)	249(99.20)	7.146	0.008^a^
Β-blocker	62(84.93)	219(87.25)	0.264	0.607
ACEIs or ARBs	24(32.88)	106(42.23)	2.060	0.151
Statin	69(94.52)	243(96.81)	0.314	0.575
Diuretics	27(36.99)	71(28.29)	2.029	0.154
Insulin treatment	4(5.48)	9(3.59)	0.150	0.699

^a^is significant

**Table 2:** Comparison of laboratory data between after hospital discharge the event and non-the event

	the event(*n = 73*)	non-the event(*n = 251*)	*t/χ2/z*	*P value*
Hemoglobin(g/L)	137(18.5)	135(21)	−1.849	0.064
Glycosylated hemoglobin(%)	6.1(1.27)	6.1(1.23)	−0.305	0.760
Glucose (mmol/L)	6.43(2.78)	6.23(2.62)	−1.122	0.262
eGFR (ml/min/1.73m^2^)	104.80(26.58)	104.76(29.96)	−0.362	0.718
Creatinine (μmol/L)	62(21)	59(23)	−1.140	0.254
UA (μmol/L)	296.50(117)	294.50(113)	−0.657	0.511
AST(U/L)	191(327)	169.50(215)	−1.461	0.144
ALT(U/L)	49(43)	45(36)	−1.637	0.102
Albumin(g/L)	38.10(5.5)	37.35(5.8)	−0.648	0.517
LDL-C (mmol/L)	3.04(1.88)	2.80(1.17)	−2.497	0.013^a^
HDL-C (mmol/L)	0.99(0.30)	1.03(0.35)	−0.489	0.625
Triglycerides (mmol/L)	1.18(0.99)	1.18(0.85)	−0.562	0.574
Total cholesterol (mmol/L)	4.31(1.53)	4.32(1.34)	−0.339	0.734
Lp(a)(mg/L)	261(207)	242(234)	−0.188	0.851
Hs-TnT (ng/L)	3433(4710.25)	3155.50(5428.75)	−0.641	0.521
NT-proBNP (pg/ml)	1091(2426.75)	661.55(1483.93)	−2.841	0.004^a^
SII (before PPCI)(T0)	1334.58(1265.52)	890(1011.97)	−3.378	0.001^a^
SII(12 h after PPCI)(T1)	1676.78(1661.00)	1107.40(1147.42)	−4.031	< 0.001^a^
SII(24 h after PPCI)	1212.70(730.09)	842.06(673.34)	−4.637	< 0.001^a^
SII (48 h after PPCI)	1044.06(601.88)	632.12(429.28)	−5.871	< 0.001^a^
SII before discharge(T2)	941.63(458.92)	584.93(403.28)	−6.282	< 0.001^a^
SII (1 month discharge)(T1M)	1005.48(580.87)	489.63(252.03)	−10.187	< 0.001^a^
cTnI_max_ (ng/ml)	4.26(7.59)	3.77(5.73)	−1.098	0.272
CK-MB_max_ (ng/ml)	184.00(248)	139.60(226)	−1.002	0.316
hs-CRP_max_ (mg/L)	20.70(31.25)	14.30(29.10)	−2.188	0.029^a^

^a^ is significant

Tables 3 and 4 summarize the demographic and laboratory data and previous treatment information for all patients. Event and non-event groups were divided based on the presence or absence of in-hospital outcomes. Compared with the non-event group, the chest pain period (h), pain-to-balloon time (h), Killip classification, stent length, glucose, cTnI_max_, hs-CRP_max_ and the five groups of SII levels were statistically significant in the event group. (*P < 0.05*).

**Table 3:** Comparison of characteristics, medications between hospitalization the event and non-the event

	The event(*n = 39*)	non-the event(*n = 285*)	*t/χ2/z*	*P value*
Gender				
Male, n(%)	33(84.62)	218(76.49)	1.297	0.255
Female, n(%)	6(15.38)	67(23.51)		
Age, n(%)	69(20)	66(17)	−0.382	0.702
Chest pain period(h)	6(5.5)	4(4)	−2.070	0.038^a^
Pain-to-balloon time(h)	7.1(6.5)	5.35(4.43)	−2.283	0.022^a^
Door-to-balloon time(h)	1(0.58)	1(0.61)	−0.785	0.433
hospitalization day(d)	6(2)	6(2)	−0.148	0.882
History				
Hypertension,n(%)	18(46.15)	117(41.05)	0.367	0.544
Diabetes mellitus,n(%)	9(23.08)	56(19.65)	0.251	0.616
Stroke or TIA,n(%)	6(15.38)	28(9.82)	0.615	0.433
Previous MI/PCI/CABG,n(%)	3(7.69)	16(5.61)	0.024	0.877
Heart Failure,n(%)	3(7.69)	7(2.46)	1.638	0.201
Coronary heart disease,n(%)	6(15.38)	29(10.18)	0.501	0.479
Current smoking status,n(%)	11(28.21)	100(35.09)	0.722	0.396
Current drinking status,n(%)	8(20.51)	52(18.25)	0.117	0.732
Admission medication				
Aspirin,n(%)	39(100.00)	285(100.00)	–	–
Clopidogrel or Tigrigrel,n(%)	39(100.00)	285(100.00)	–	–
ACEIs or ARBs,n(%)	19(48.72)	164(57.54)	1.087	0.297
CCB,n(%)	16(41.03)	135(47.37)	0.555	0.456
Β-blocker,n(%)	34(87.18)	254(89.12)	0.008	0.928
Statin,n(%)	39(100.00)	285(100.00)	**–**	–
Diuretics,n(%)	18(46.15)	95(33.33)	2.483	0.115
SBP (mmHg)	119(30)	123(29)	−1.840	0.066
DBP (mmHg)	72(14)	78(18)	−1.544	0.123
Heart-rate (beats-per-minute)	83.28 ± 14.84	80.70 ± 14.10	0.018	0.287
Killip class,n(%)				
1–2	30(76.92)	263(92.28)	7.661	0.006^a^
3–4	9(23.08)	22(7.72)		
Target Vessel,n(%)				
LM	3(7.69)	6(2.11)	2.166	0.141
LAD	25(64.10)	166(58.25)	0.486	0.486
LCX	6(15.38)	31(10.88)	0.315	0.574
RCA	7(17.95)	88(30.88)	2.767	0.096
Simultaneous treatment of vascular situations,n(%)				
1	37(94.87)	279(97.89)	0.349	0.555
> 1	2(5.13)	6(2.11)		
Vessel-disease (stenosis > 50%)				
1 vessel,n(%)	11(28.21)	79(27.72)	0.004	0.949
2 vessels,n(%)	15(38.46)	66(23.16)	3.508	0.061
3 vessels,n(%)	13(33.33)	140(49.12)	3.432	0.064
PCI type,n(%)				
Only PTCA	5(12.82)	30(10.53)	0.025	0.875
PTCA and Stent	34(87.18)	255(89.47)		
Stent type,n(%)				
Drug eluting stent	10(25.64)	119(41.75)	3.717	0.054
Bare metal stent	29(74.36)	166(58.25)		
Number of stents inserted,n(%)				
1 piece	32(82.05)	261(91.58)	2.582	0.108
≥2 piece	7(17.95)	24(8.42)		
Stent length (mm)	28(6)	23(11)	−1.995	0.046^a^
Stent Diameter (mm)	3(0.25)	3(0.5)	−0.531	0.595
IVUS Assist,n(%)				
Yes	3(7.69)	10(3.51)	0.662	0.416
No	36(92.31)	275(96.49)		
TIMI after PPCI n(%)				
3	37(94.87)	279(97.89)	0.349	0.555
<3	2(5.13)	6(2.11)		
site of AMI n(%)				
anterior	25(64.10)	145(50.88)	6.695	0.112
antero-septal	1(2.56)	9(3.16)		
high side wall	2(5.13)	3(1.05)		
Inferior wall	11(28.21)	125(43.86)		
anterolateral	0(0.00)	3(1.05)		

^a^is significant

**Table 4:** Comparison of laboratory data between hospitalization the event and non-the event

	The event(*n = 39*)	non-the event(*n = 285*)	*t/χ2/z*	*P value*
Hemoglobin(g/L)	138(31)	135(20.50)	−0.460	0.645
Glycosylated hemoglobin(%)	6.2(1.70)	6.1(1.20)	−0.308	0.758
Glucose (mmol/L)	6.49(2.96)	6.20(2.64)	−2.034	0.042^a^
eGFR (ml/min/1.73m^2^)	104.89(34.78)	104.71(28.31)	−0.445	0.656
Creatinine (μmol/L)	60(17)	59(23)	−0.742	0.458
UA (μmol/L)	294(114)	295(115)	−0.658	0.511
AST(U/L)	238(365)	173(211)	−1.359	0.174
ALT(U/L)	53(45)	46(37)	−1.939	0.053
Albumin(g/L)	38.10(4.70)	37.30(5.90)	−0.672	0.502
LDL-C (mmol/L)	2.83(1.13)	2.78(1.22)	−0.582	0.560
HDL-C (mmol/L)	1.03(0.38)	1.02(0.34)	−0.773	0.440
Triglycerides (mmol/L)	1.26(0.99)	1.17(0.88)	−0.699	0.485
Total cholesterol (mmol/L)	4.34(1.10)	4.29(1.43)	−0.442	0.658
Lp(a)(mg/L)	215(258)	246(217)	−0.136	0.892
Hs-TnT (ng/L)	4622(8805)	3150(4889)	−1.170	0.242
NT-proBNP (pg/ml)	933.70(2264.30)	729.30(1530.93)	−1.164	0.245
SII(before PPCI)(T0)	1477.50(1500.91)	901.28(996.63)	−3.375	0.001^a^
SII(12 h after PPCI)(T1)	2779.67(2189.23)	1067.95(997.54)	−8.031	< 0.001^a^
SII(24 h after PPCI)	1483.76(1834.28)	872.23(664.46)	−4.295	< 0.001^a^
SII (48 h after PPCI)	1074.46(891.21)	679(466.88)	−4.622	< 0.001^a^
SII before discharge(T2)	1000.53(501.99)	624(448.04)	−5.461	< 0.001^a^
cTnI_max_ (ng/ml)	5.90(7.19)	3.60(5.66)	−2.587	0.010^a^
CK-MB_max_ (ng/ml)	195.90(235)	143.00(214)	−1.748	0.080
hs-CRP_max_ (mg/L)	22.10(89.40)	14.50(28.05)	−3.160	0.002^a^

^a^is significant

In the multivariable logistic regression model, IVUS assistance, LDL-C level, and SII at 1 month (T1M)(*OR: 0.05, 1,95%, CI: 0.010–0.251, P < 0.001; OR: 1.890, 95%, CI: 1.214–2.941, P = 0.005; OR: 1.008, 95%, CI: 1.006–1.010, P < 0.001*), predict the out-of-hospital outcomes, respectively (Table 5). Additionally, SII 12 h after (T1), cTnI_max_, and hs-CRP_max_ (*OR: 1.001, 95%, CI: 1.001–1.001, P < 0.001; OR: 1.075, 95%, CI: 1.008–1.148, P = 0.029; OR: 1.008, 95%, CI: 1.002–1.015, P = 0.012*) respectively, were independently associated with in-hospital outcomes (Table 6).

**Table 5:** Univariable and multivariable logistic regression analysis for independent predictors of out-of hospital outcomes

Variable	Univariate		Multivariate	
	*OR (95%CI)*	*P value*	*OR (95%CI)*	*P value*
2Vessal diseases(> 50%)	0.551(0.313–0.973)	0.040	0.854(0.297–2.457)	0.769
3Vessal diseases(> 50%)	1.717(1.005–2.934)	0.048	1.612(0.620–4.189)	0.327
Stent number	0.306(0.143–0.657)	0.002	0.462(0.125–1.703)	0.246
IVUS assisted	0.231(0.075–0.710)	0.011	0.051(0.010–0.251)	< 0.001^*^
P2Y12 receptor inhibitors	9.154(1.738–48.223)	0.009	2.844(0.296–27.287)	0.365
LDL-C	1.550(1.195–2.011)	0.001	1.890(1.214–2.941)	0.005^*^
NT-proBNP	1.000(1.000–1.001)	0.569	–	–
SII(1 month discharge)(T1M)	1.007(1.005–1.009)	< 0.001	1.008(1.006–1.010)	< 0.001^*^
Age	0.990(0.970–1.010)	0.327	–	–
HDL-C	0.795(0.280–2.257)	0.666	–	–
cTnImax	1.020(0.974–1.068)	0.412	–	–
CK-MBmax	1.001(0.999–1.003)	0.318	–	–
hs-CRPmax	1.005(1.000–1.010)	0.037	1.006(0.998–1.015)	0.145

^*^is significant

**Table 6:** Univariable and multivariable logistic regression analysis for independent predictors of in-hospital outcomes

Variable	Univariate		Multivariate	
	*OR (95%CI)*	*P value*	*OR (95%CI)*	*P value*
Age	1.004(0.978–1.030)	0.786	–	–
Chest pain period	1.075(1.001–1.155)	0.048	1.108(0.899–1.365)	0.335
Pain-to-balloon time	1.070(1.005–1.139)	0.033	1.005(0.839–1.203)	0.959
Killip class> 2	0.279(0.118–0.661)	0.004	0.554(0.168–1.821)	0.330
Stent length	1.054(1.000–1.111)	0.052	–	–
Glucose	1.039(0.938–1.151)	0.464	–	–
SII(12 h after PPCI)(T1)	1.001(1.001–1.001)	< 0.001	1.001(1.001–1.001)	< 0.001^*^
cTnI_max_	1.053(1.001–1.109)	0.045	1.075(1.008–1.148)	0.029^*^
CK-MB_max_	1.002(1.000–1.005)	0.079	–	–
hs-CRP_max_	1.011(1.006–1.017)	<0.001	1.008(1.002–1.015)	0.012^*^

^*^is significant

### The overall levels of change in blood SII and hs-CRP levels at different onset times

**Fig. 2:**
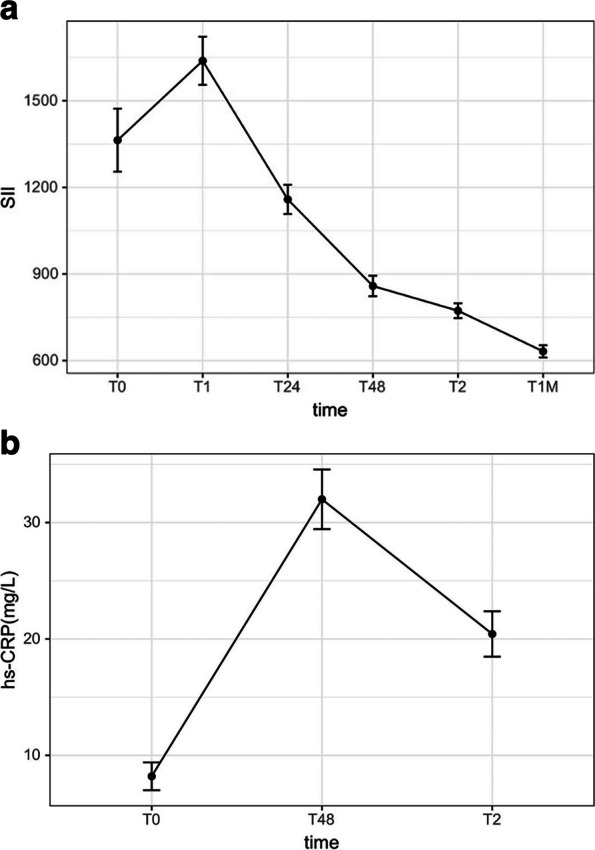
**a**. Six groups of SII levels series trend graph. **b**. Three groups of hs-CRP levels series trend graph

The time profile showed that the SII started to rise before PPCI (T0), peaked at 12 h after PPCI, and then gradually decreased to a valley level 1 month after discharge. The hs-CRP level started to rise before PPCI (T0), gradually increased to a peak at 48 h after PPCI, and then rapidly decreased. In addition, we saw that the time of SII_max_ level appeared earlier than hs-CRP_max_ (Fig. 2(a), (b)).

### Predictive value of ROC curve analysis of SII levels, hs-CRP_max_, cTnI_max_, CK-MB_max_ for the occurrence of outcomes both hospitalization and discharge after PPCI in STEMI patients

**Fig. 3:**
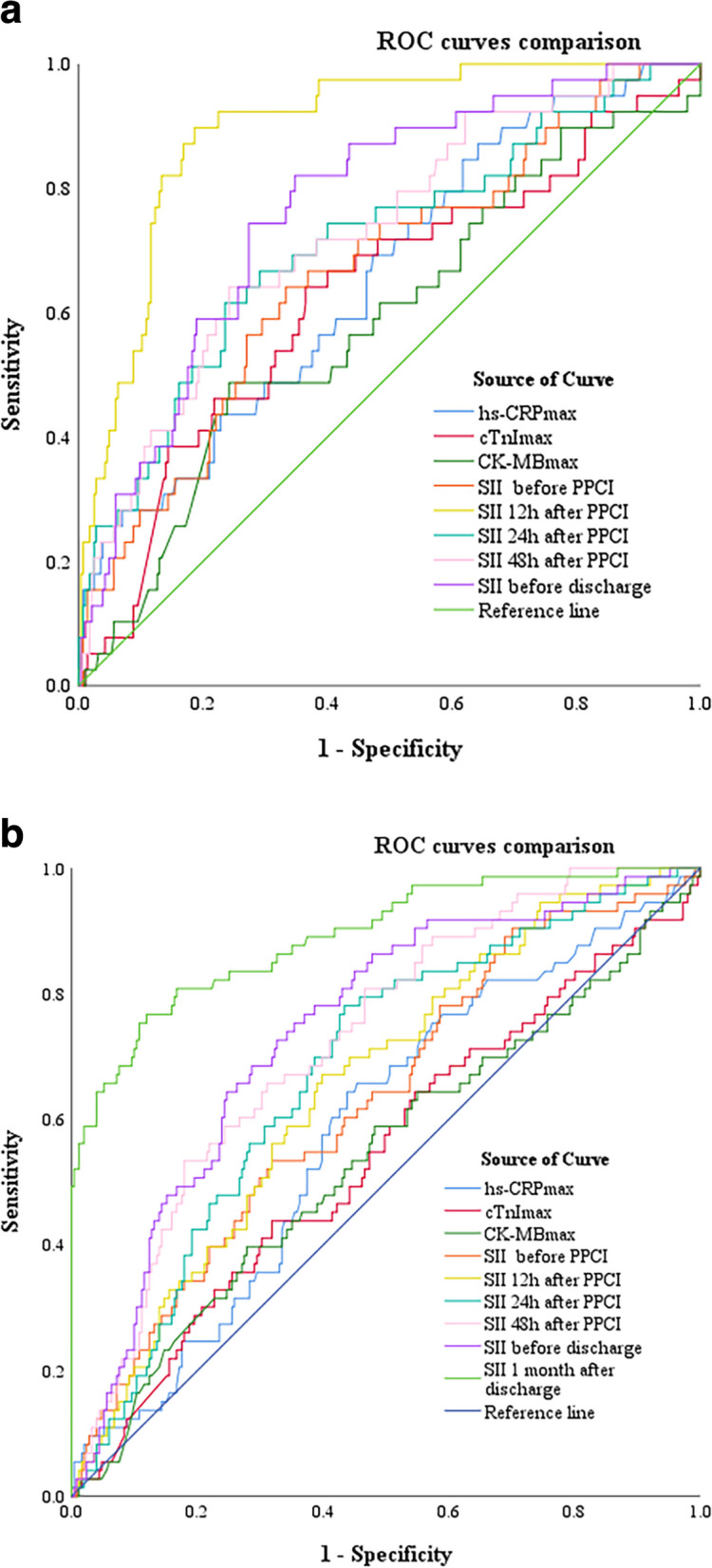
**a** Receiver-operating characteristic (ROC) curve analyses of five groups of SII, hs-CRP_max_, cTnI_max_, CK-MB_max_ for predicting in-hospital outcomes in STEMI patients. **b**. Receiver-operating characteristic (ROC) curve analyses of six groups of SII, hs-CRP_max_, cTnI_max_, CK-MB_max_ for predicting out-of hospital outcomes in STEMI patients

To predict in-hospital and out-of-hospital outcomes, receiver operating characteristic (ROC) curves of six groups of SII, hs-CRP_max_, cTnI_max,_ and CK-MB_max_ were plotted (Fig. 3(a), (b)). The optimal critical values, sensitivities, and specificities were calculated (Tables 7 and 8). In-hospital ROC analysis illustrated that the best cutoff value of the SII (T1) to predict outcomes during hospitalization was 1915.77 with 89.70% sensitivity and 81.40% specificity (*AUC:0.896; 95%, CI: 0.852–0.941; P < 0.001*). The discriminative value of SII 12 h after PPCI was higher than that of the others. Out-of-hospital ROC analysis indicated that the best cut-off value of the SII 1 month, to predict the outcomes of discharge was 696.43 with 76.70% sensitivity and 88.00% specificity (*AUC: 0.892; 95%, CI: 0.846–0.937; P < 0.001*). The discriminative value of the SII at 1 month after discharge was the highest.

**Table 7:** Receiver-operating characteristic (ROC) curve analyses of five groups of SII, hs-CRP_max_, cTnI_max_, CK-MB_max_ for predicting in-hospital outcomes in STEMI patients

	*Cut-off*	*Sensitivity*	*Specificity*	*AUC*	*95%CI*	*P value*
SII before(T0)	1264.15	0.641	0.667	0.667	0.575–0.758	0.001^*^
SII12h after(T1)	1915.77	0.897	0.814	0.896	0.852–0.941	<0.001^*^
SII24h after	1282.53	0.615	0.765	0.712	0.620–0.804	<0.001^*^
SII48h after	956.76	0.641	0.758	0.728	0.644–0.812	<0.001^*^
T2	779.79	0.821	0.653	0.770	0.697–0.843	<0.001^*^
hs-CRP_max_	9.050	0.872	0.358	0.656	0.566–0.746	0.002^*^
cTnI_max_	4.887	0.641	0.635	0.628	0.529–0.726	0.010^*^
CK-MB_max_	276.11	0.487	0.758	0.586	0.488–0.685	0.080

^*^is significant. (AUC, area under the curve; ROC, receiver operating characteristic; SII, systemic immune-inflammation)

**Table 8:** Receiver-operating characteristic (ROC) curve analyses of six groups of SII, hs-CRP_max_, cTnI_max_, CK-MB_max_ for predicting out-of hospital outcomes in STEMI patients

	*Cut-off*	*Sensitivity*	*Specificity*	*AUC*	*95%CI*	*P value*
SII before(T0)	1278.35	0.534	0.681	0.630	0.558–0.702	0.001^*^
SII12h after(T1)	1361.13	0.671	0.602	0.655	0.587–0.723	<0.001^*^
SII24h after	918.45	0.781	0.566	0.678	0.612–0.745	<0.001^*^
SII48h after	1055.28	0.534	0.821	0.726	0.664–0.787	<0.001^*^
T2	735.32	0.753	0.645	0.742	0.679–0.804	<0.001^*^
SII1month(T1M)	696.43	0.767	0.880	0.892	0.846–0.937	<0.001^*^
hs-CRP_max_	16.45	0.644	0.562	0.584	0.512–0.657	0.029^*^
cTnI_max_	5.668	0.438	0.681	0.542	0.465–0.619	0.272
CK-MB_max_	252.70	0.397	0.721	0.539	0.460–0.617	0.316

^*^is significant. (AUC, area under the curve; ROC, receiver operating characteristic; SII, systemic immune-inflammation)

### Correlation of high and low SII level groups with cumulative all-cause mortality in STEMI patients after PPCI

**Fig. 4:**
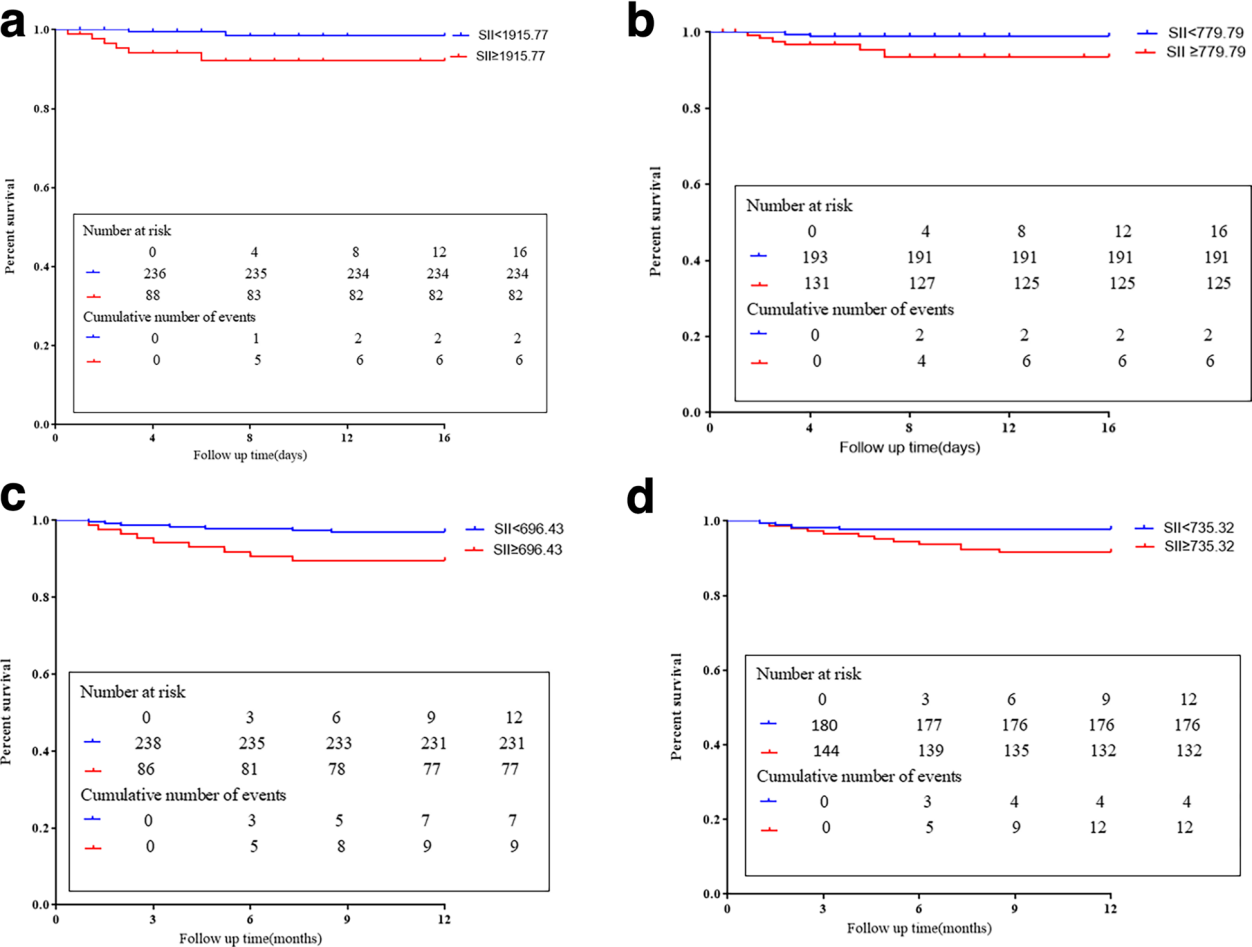
**a** Kaplan-Meier Survival Curves for All-cause Mortality during hospitalization according to SII 12 h after PPCI(T1). **b** Kaplan-Meier Survival Curves for All-cause Mortality during hospitalization according to SII before hospital discharge(T2). **c**. Kaplan-Meier Survival Curves for All-cause Mortality after hospital discharge according to SII 1 month after discharge(T1M). **d**. Kaplan-Meier Survival Curves for All-cause Mortality after hospital discharge according to SII before hospital discharge(T2)

There was the occurrence of in-hospital and out-of-hospital outcomes (Tables 9, 10). Kaplan-Meier curves were used to analyze the differences in survival rates at different SII inflammation levels according to T1, T2, and T1M during hospitalization and after hospital discharge (Fig. 4 (a)–(d)).

**Table 9:** Occurrence of cardiovascular events during hospitalization in STEMI patients after PPCI

In-hospital course	*n(%)*
Cardiogenic shock	4(1.23%)
Acute respiratory failure	2(0.62%)
Acute kidney injury	4(1.23%)
Ventricular arrhythmia	13(4.01%)
Recurrent myocardial infarction	5(1.54%)
Repeat revascularization	3(0.93%)
All-cause mortality	8(2.47%)
Combined	39(12.04%)

**Table 10:** Occurrence of cardiovascular events after hospital discharge in STEMI patients after PPCI

Out-hospital course	*n(%)*
All-cause mortality	16(4.94%)
Recurrent myocardial infarction	17(5.25%)
Repeat revascularization	28(8.64%)
Ventricular arrhythmia	12(3.70%)
Combined	73(22.53%)

### Kaplan-Meier survival curves for all-cause mortality during hospitalization according to SII 12 h after PPCI (T1) and SII before hospital discharge (T2)

The upper panel shows patients with a critical SII (T1) below or above 1915.77. The lower figure showed 779.79 at T2 (h). SII value <cutoff (blue curve), values ≥cutoffs (red curve). The functions in T1 (*P = 0.0017*) and T2 (*P = 0.0354*) showed that patients with an SII value below (blue curve) had a significantly higher cumulative survival than patients with an SII cutoff value above (red curve) (Fig. 4 (a), (b)).

### Kaplan-Meier survival curves for all-cause mortality discharge according to SII 1 month after discharge and SII before discharge(T2)

The upper panel showed patients with a critical SII value (T1M) below or above 696.43. The lower panel showed 735.32 at T2. SII value <cut-off (blue curve), values ≥cutoffs (red curve). The SII-value (SII 1 month after) (*P = 0.0025*) and T2 (*P = 0.0124*) showed that patients with an SII value below (blue curve) had significantly higher cumulative survival than those with SII cut-off values above (red curve) (Fig. 4 (c), (d)).

## Discussion

This retrospective analysis found that the SII, a novel inflammatory marker, was associated with the occurrence of hospitalization and discharge outcomes in patients with acute STEMI after PPCI. Between the event and non-event groups, SII levels responded differently to high and low inflammatory statuses, which had an impact on prognosis. Patients with a highly inflammatory state have a relatively poor prognosis.

Inflammation plays a key role in the development of atherosclerosis, acute myocardial infarction, and poor prognosis after myocardial infarction. Plaque rupture triggers an inflammatory response, which releases highly thrombogenic components and promotes thrombus formation [16]. In the acute phase of myocardial infarction, the inflammatory response becomes significant with acute exacerbation, and the higher the degree of inflammation, the larger the area of myocardial ischemic necrosis. The COLCOT trial showed that the use of low-dose colchicine within 3 days after myocardial infarction (MI) reduced the occurrence of ischemic cardiovascular events. Patients can benefit from the early in-hospital use of colchicine after MI [17]. This suggests that the occurrence of MI is associated with inflammatory involvement and that early suppression of inflammation after MI provides even greater benefits.

Leukocytes and their subtypes, such as neutrophils, monocytes, lymphocytes, and platelets, are important inflammatory cells in the circulation and have been shown in several studies to be associated with the prognosis of patients with acute MI. Leukocyte count is an independent predictor of AM I[18], and elevated leukocyte levels are associated with increased mortality in patients with STEMI [19]. During inflammation, pre-stimulated neutrophils infiltrating the vessel wall release reactive oxygen species, cytokines, and myeloperoxidases that damage the vascular endothelium to promote inflammation progression. Elevated neutrophil counts are associated with a high risk of ischemic events [20] and AMI mortality [21]. In the inflammatory state of AMI, cortisol levels are elevated and lymphocytes are affected by cortisol levels, resulting in decreased lymphocyte levels [22]. Lymphocytopenia was independently associated with mechanical complications and mortality in patients with acute STEMI [23]. Monocytes, a basic component of the immune system, are considered to be predictors of coronary events [24], and an increase in their number has been shown to correlate with the prognosis of AMI [25]. Platelets play a role in inflammatory and immune responses through the release of pro-inflammatory cytokines and interactions with endothelial cells, leukocytes, and smooth muscle cells [26, 27] and are significantly associated with inflammation and atherothrombosis [28].

Monitoring several inflammatory markers in peripheral blood has been proposed to assess cardiovascular risk in patients with acute myocardial infarction. Markers such as the NLR, PLR, and LMR have been introduced as new markers. High NLR was significantly associated with cardiovascular and all-cause mortality in patients with ST-segment elevation myocardial infarction during hospitalization or in the long term [29, 30]. Low LMR correlates with poor outcomes in patients [31]. PLR is associated with long-term mortality in STEMI patients [32]. Recently, Hu et al. developed an innovative predictive marker called the SII based on a prospective cohort study [33]. SII is a novel inflammatory parameter calculated as (N × P)/L (N, P, and L represent the neutrophil count, platelet count, and lymphocyte count, respectively) and represents three important immune response pathways: inflammatory response, thrombosis, and organismal stress response. It is a prognostic indicator of poor outcomes in various types [34, 35]. Its study in cardiovascular disease has also been conducted. SII has been found to predict clinical outcomes in patients with coronary artery disease [36]. Su et al. [37] demonstrated that a high SII was independently associated with all-cause mortality at 30 days, 90 days, and 1 year in patients with acute coronary syndrome. Öcal et al. [15] demonstrated that the SII was independently associated with all-cause mortality and adverse cardiovascular events during hospitalization and at 3-year follow-up after PPCI in patients with acute STEMI. Additionally, other studies have shown that the SII may indicate short- and long-term mortality in patients with heart failure (HF) with reduced ejection fraction (HFrEF) and acute type A aortic dissection (ATAD) undergoing surgery and with infective endocarditis [38–40]. SII can also be used as an independent predictor of NOAF following STEMI [41]. All these studies used a single SII level at baseline and illustrated that higher SII levels were associated with a poorer prognosis.

Our study differs from the above studies in that we assessed the process of dynamic changes in SII levels. The process of inflammatory activity is dynamic. During the intraoperative and postoperative periods, the stent, as a foreign object, stimulates endothelial cells and increases inflammatory radical responses, which may manifest as an increase in inflammatory indices. During hospitalization and after hospital discharge, the patients were treated with appropriate antiplatelet and lipid-regulating drugs, which also affected the inflammatory process. Therefore, dynamic changes in SII levels can better reflect the overall situation than a single SII level. In our study, we attempted to determine whether dynamic changes in the SII were related to in-hospital and out-of-hospital outcomes in patients with STEMI undergoing PPCI. This study analyzed the relationship between serial changes in the SII during the perioperative period of PPCI and the occurrence of the primary endpoint. The results showed that high SII levels were independently associated with the primary endpoint during the postoperative follow-up period of patients (*P < 0.05*). In contrast, patients with high SII had lower survival rates than those with low SII. By dynamically analyzing SII levels in six groups, hs-CRP_max_, cTnImax, cTnI_max_, CK-MB_max,_ and constructing ROC curves at each node, the analysis showed that the SII 12 h after PPCI (T1) and 1 month after hospital discharge (T1M) had excellent predictive value for the occurrence of in-hospital and out-of-hospital outcomes. This can be attributed to two reasons. First, inflammation may be closely related to the prognosis of in-hospital outcomes in patients with STEMI due to the intense inflammatory response during the AMI phase. Second, 1 month after hospital discharge, most patients benefited from the appropriate use of antiplatelet and lipid-regulating drugs, which can improve the local inflammatory state of the myocardium, while a small number of patients still had local myocardial inflammation, thus leading to different prognoses. This inspired us to focus on peak SII levels during hospitalization. When the peak in-hospital SII was > 1915.77, timely intervention was required to effectively reduce in-hospital outcomes. Meanwhile, focusing on the lowest level of the SII at 1 month after hospital discharge and maintaining it below 696.43 can effectively reduce the occurrence of out-of-hospital outcomes. Thus, this study provides guidance for improving patient prognosis.

However, as there are few studies on the correlation between dynamic changes in SII levels during the perioperative period of PPCI and primary endpoint occurrence in patients with STEMI, several limitations were present: 1. This was a single-center, retrospective study with a small sample size. These conclusions may have been affected by a selection bias. 2. Other conventional factors that respond to the inflammatory status, such as calcitonin, IL-6, and myeloperoxidase, were not included in this study. 3. Although independent risk factors were identified by multivariate regression, some undefined factors remained, which affected the study results. We believe that future prospective multicenter studies with large sample sizes could confirm the inflammatory status to meet the control standard and thus reduce major cardiovascular events, similar to the application of lipid-regulating medications to lower lipid levels and bring lipids into general standards.

## Conclusion

This study obtained important prognostic information from the normal blood tests of patients with STEMI who underwent PPCI. This study showed that dynamic changes in the SII in patients with STEMI during the perioperative period of PPCI were correlated with the occurrence of in-hospital and out-of-hospital outcomes. SII is a simple and practical indicator for identifying high-risk patients after PPCI. Based on the trend of serial changes in SII levels, preventive measures can be taken in patients with high inflammatory status to reduce the occurrence of cardiovascular events**.**

### Supplementary Information

**Additional file 1.**

## Data Availability

Raw data supporting the conclusions of this article were provided by the author (AL) without unnecessary reservations. Additional information on this article had been added to the attachment.
